# 

**Published:** 1903-11-14

**Authors:** 


					THE HOSPITAL. "The Standard of highest purity."?Lancet. November 14th, 1903.
CADBURY'S If CADBURY'S
COCOA |L JEH Jet - COCOA
'* A PERFECT FOOD.'
HO DRUGS OR ALKALIK?^UfPlX
Em
/
s'aarr Western Infirmary
No. ?9/1. Vol. XXXY. [aNewspfp^] SATURDAY,
Nov. 14th, 1903.
[SmDSCBreePp.0n2erms'] PRICE THREEPENCE.

				

